# Design and Optimization of a Linear Wavenumber Spectrometer with Cylindrical Optics for Line Scanning Optical Coherence Tomography

**DOI:** 10.3390/s21196463

**Published:** 2021-09-28

**Authors:** Sevin Samadi, Javad Dargahi, Sivakumar Narayanswamy

**Affiliations:** Department of Mechanical, Industrial and Aerospace Engineering (MIAE), Concordia University, Montreal, QC H3G 1M8, Canada; javad.dargahi@concordia.ca (J.D.); siva.narayanswamy@concordia.ca (S.N.)

**Keywords:** optics, spectrometer, line scanning OCT, linear wavenumber

## Abstract

We report the design of a high-efficiency spectral-domain spectrometer with cylindrical optics for line scanning optical coherence tomography (OCT). The spectral nonlinearity in k space (wavenumber) lowers the depth-dependent signal sensitivity of the spectrometers. For linearizing, in this design, grating and prism have been introduced. For line scanning, a cylindrical mirror is utilized in the scanning part. Line scanning improves the speed of imaging compared to fly-spot scanning. Line scanning OCT requires a spectrometer that utilizes cylindrical optics. In this work, an optical design of a linear wavenumber spectrometer with cylindrical optics is introduced. While there are many works using grating and prism to linearize the K space spectrometer design, there is no work on linearizing the k-space spectrometer with cylindrical optics for line scanning that provides high sensitivity and high-speed imaging without the need for resampling. The design of the spectrometer was achieved through MATLAB and ZEMAX simulations. The spectrometer design is optimized for the broadband light source with a center wavelength of 830 ± 100 nm (8.607 μm${}^{-1}$− 6.756 μm${}^{-1}$ in k-space). The variation in the output angle with respect to the wavenumber can be mentioned as a nonlinearity error. From our design results, it is observed that the nonlinearity error reduced from 147.0115 to 0.0149 Δθ*μm within the wavenumber range considered. The use of the proposed reflective optics for focusing reduces the chromatic aberration and increases image quality (measured by the Strehl ratio (SR)). The complete system will provide clinicians a powerful tool for real-time diagnosis, treatment, and guidance in surgery with high image quality for in-vivo applications.

## 1. Introduction

There is considerable interest in designing ultra-broadband optical coherence tomography, which is a low coherence interferometric 3D imaging technique that provides cross-sectional views of the subsurface microstructure of biological tissue with micrometer resolution for clinical studies [1,2]. The advantage of OCT in comparison with other techniques is that the resolution is in the range of micrometers, while the depth scan range is in the order of a few millimeters [1,3,4]. Spectral-domain optical coherence tomography (SD-OCT) is used for applications in high-speed biomedical imaging. In SD-OCT, the sensitivity of the signal drops in deeper regions, and it results in lower resolution in the image. Reducing the sensitivity fall-off is the main concern in the design of the spectrometer [5,6,7,8]. Nonlinear sampling of the interferograms in wavenumber (k) space reduces the depth-dependent signal sensitivity in SD-OCT. To address this, a linear k space spectrometer with cylindrical optics for line scanning [9] is introduced. In SD-OCT, the sample is illuminated by a broadband light source. The light’s interference that is partially reflected from layers of the sample and the reference mirror is analyzed with a spectrometer. In SD-OCT, the depth profile is constructed by inverse Fourier transform (FT) of the interferograms, and it is mandatory to rescale the output from the wavelength to the wavenumber space. However, the diffraction angle of light from the grating is nonlinear with respect to the wavenumber (k). This nonlinearity will lead to two problems. First, the axial point spread function (intensity profiles emitted from the backscattered light from multiple layers of the sample along the depth [10]) will be broadened. Second, the wavenumber bandwidth integrated by an individual pixel is nonuniform. This leads to sensitivity fall-off along the depth [5,6,7,8]. To overcome this, one possibility is to increase the number of pixels in the detector [11,12]. There is often a trade-off between the pixel number and the pixel size under a limited array dimension. The increasing number of pixels will result in the reduction of the pixel size. Smaller pixels cause high distortion. In this case, the dispersed spectrum’s point spread function (PSF) plays an essential role in effective spectral sampling. If the PSF is larger than the pixel pitch, the spectrometer’s optical performance will become a limit for effective spectral sampling [13,14]. Moreover, both the system cost and the time for data analysis increase exponentially with an increasing number of pixels.

Alternatively, to reduce the signal sensitivity fall-off, the linear k-space spectrometer has been introduced [5,13]. This spectrometer uses a prism to offset the nonlinearity caused by the grating. In SD-OCT, a proper design of grating and prism can ensure linearity. While there are works on linearizing the spectrometer using prism and grating separately, in the literature, you can also find works related to “Grism” [15], where the grating is attached to the prism. The first method provides more design freedom for angular adjustments. In [14], the center wavelength is 1310 nm, and the bandwidth is 68 nm. In [8], two systems were designed, the first one has a center wavelength of 1270 nm with a bandwidth of 70 nm, and the second one has a center wavelength of 830 nm with a bandwidth of 40 nm. In these works, linearizing the k space spectrometer shows a significant improvement in signal sensitivity [7,8]. There is an increasing demand for ultra broadband OCT systems with a higher axial resolution to distinguish smaller structures [16]. It causes more difficulties for k-space spectrometer design in the nonlinearity error correction and the aberration correction for focusing lens. The axial resolution of the OCT improves as the spectral bandwidth of the source increases. While the references mentioned earlier work on smaller bandwidths, there are few works in medical imaging using broadband OCT [17,18]. In [13], an ultra broadband linear k space is introduced. However, it is based on a fly spot scanning system, and it utilizes lenses as focusing optics. The effective focal length (EFL) of the lens varies as a function of the wavelength [19]. Consequently, the lens-focused scanning system’s imaging quality is reduced due to the chromatic aberration [20]. Furthermore, the imaging quality of flying spot scanning is also reduced because of the distortion errors and motion artifacts, such as eye or body motion [21,22]. In line-scanning OCT (LS-OCT), cylindrical optics are used to focus the collimated beam as a line on the sample. Two-dimensional cross-sectional imaging data can be obtained using line-focused scanning with SD acquisition without requiring a mechanical scanner [9].

LS has a significant impact on 3D imaging. It makes 3D imaging possible by integrating a single-axis scanner. In our earlier articles [9,23,24,25,26], the optical design of a cylindrical mirror-based scanning system has been reported. A cylindrical mirror is used for focusing, and a flat mirror is used for the scanning part. In this system, an 830 nm center wavelength laser source with a spectral bandwidth of 200 nm is used, by rotation of scanning mirror ±2.4°, a 2 mm field is scanned. The idea of a line space spectrometer arises from the line scanning system of our proposed SD-OCT [23]. This spectrometer can resolve data from an illumination incident with a line width of 2 mm × 40 μm in a single scan. The literature review shows there have been works on linearizing spectrometers, while there are few works on linearizing the ultra broadband spectrometers [8,27]. Still, there has been no work on reflective optics focusing and line scanning. Considering the advantages of line scanning and reflective focusing, the predominant contribution of this work is to design and optimize the linear k space spectrometer for mirror focused line scan OCT.

Two linear k space line scanning spectrometer models using a combination of grating and prism are introduced in this work. One of them is based on the transmission grating, and the second one is based on the reflective grating [17]. These two systems are compared based on their optical performance. All spectrometers were designed and implemented using optical studio ZEMAX. In addition, MATLAB was used for proof of the design. The use of the proposed all-reflective components reduces the chromatic aberration and increases image quality (measured by the Strehl ratio (SR)). The complete system will allow clinicians a powerful tool for real-time diagnosis, treatment, and guidance in surgery with high image quality for in-vivo applications.

## 2. Theory and Properties

**Figure 1:**
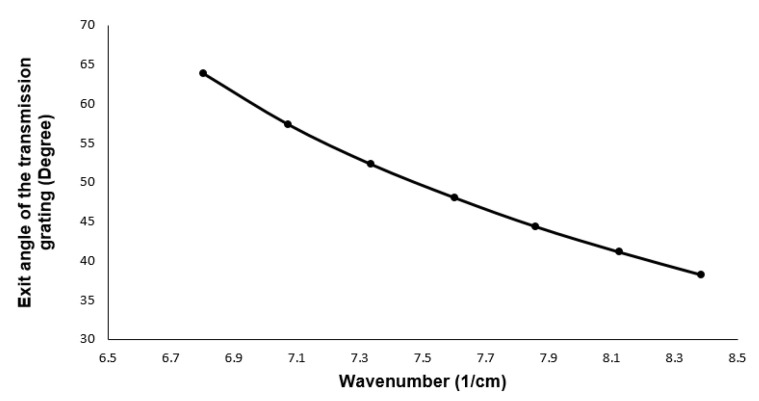
Exit angle of the light from the grating vs. wavenumber.

The relationship between the exit angle and the transmission and reflective gratings wavelength is shown in Equation (1).

(1) $$d[\sin\left(\theta_{i}\right)\pm \sin\left(\theta_{m}\right)]=m\lambda$$

where *d* is the grating period, $\theta_{m}$ is the diffraction angle, $\theta_{i}$ is the incident angle, *m* is the diffraction order, and λ is the wavelength. In this equation, the + is for the reflective, and − is for the transmission grating. Based on this equation, it is clear that the wavenumber’s relationship with the diffraction angle is nonlinear. Figure 1 shows this relationship for a ‘*d*’ of 1600 lines/mm, ‘*m*’ of 1, and λ, which varies between 730 and 930 nm (8.607 μm${}^{-1}$− 6.756 μm${}^{-1}$ in k-space).

**Figure 2:**
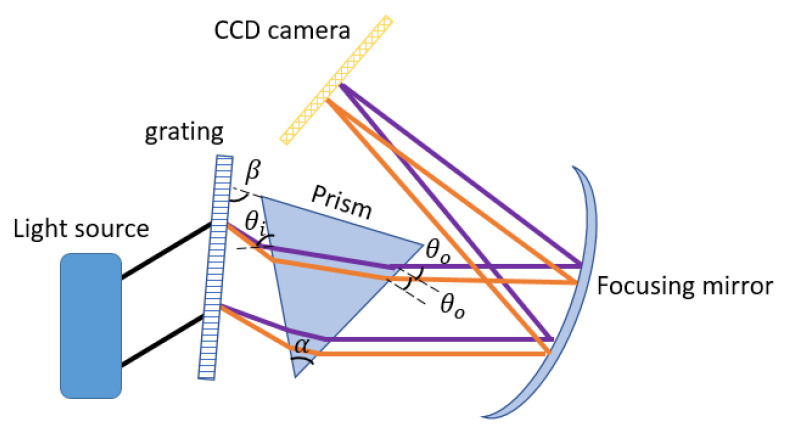
Schematic of the spectrometer using grating and prism (two wavelengths, orange longer and purple shorter).

**Figure 3:**
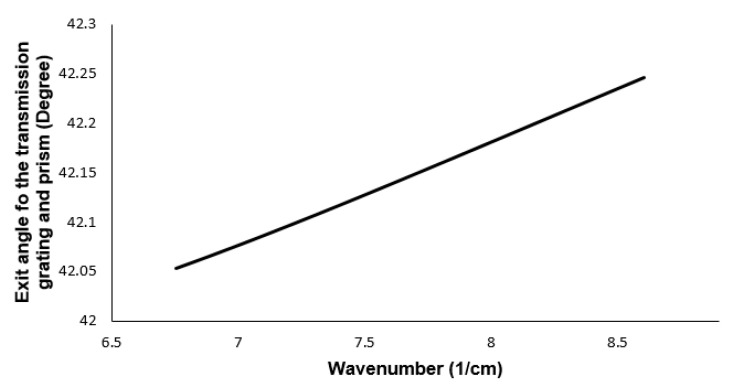
The variation of the exit angle for different wavenumbers for combination of transmission grating and prism.

The mathematical proof that prism and grating at specific angles can ensure the linearity of the output angle will be presented in this work. This linearity will reduce sensitivity fall-off and computing time. A prism will be inserted in the proposed design between the grating and the focusing mirror, as shown in Figure 2. If the prism and grating angles are set up such that the center wavelength will propagate parallel to the base of the prism, linearity can be ensured [14]. The linearity of the output angle with respect to the wavenumber can be expressed using Equation (2) and combining Snell’s law with geometry. The angle after the dispersion group for each wavenumber *k* can be expressed by Equation (3) [28].

(2) $$\theta_{i}\left(\lambda \right)=\arcsin\left(n\left(\lambda_{c}\right)\cos\left(\alpha \right)\right)+\arcsin\left(d\lambda -0.5d\lambda_{c}\right)-\arcsin\left(0.5d\lambda_{c}\right)$$

(3) $$\theta_{o}=\arcsin\left(n_{k}\left(\sin\left(\alpha -\arcsin\left(\frac{\sin(\theta_{k})-\beta }{n_{k}}\right)\right)\right)\right)$$

where *d* is the grating period, *n* is the wavelength-dependent refractive index of the prism, $n_{k}$ is the wavenumber dependent refractive index, and $\theta_{k}$ is the diffracted angle of each wavenumber. $\theta_{i}$ is the incident angle to the prism, which is related to the wavelength, refractive index and apex angle of the prism, $\theta_{o}$ is the angle out of the prism. α is the base angle of the prism, and $\lambda_{c}$ is the center wavelength of the light source. $\theta_{k}$ is the diffracted angle of each wavenumber, $\sin\theta_{k}=2\pi /kd-\sin\theta_{i}$ [28]. The α is fixed at 35${}^{\circ }$, and d is fixed at 1600 lines/mm. For any given α and d, each material has a particular value of β. $\theta_{o}$ is a function of k, and for different wavenumbers, the variation of the $\theta_{o}$ should remain minimal in Equation (3). For the wavelengths range considered, β of 25${}^{\circ }$ provides the least variation in $\theta_{o}$ in Equation (3). The angular variation must be constant to ensure linearity in the k-space. Comparison of the exit angle out of the prism and grating with respect to the wavenumber is shown in Figure 3. The variation of output angle while using only the grating is also shown in the figure. The angular variation for the transmission grating is between 64${}^{\circ }$ and 38${}^{\circ }$ for the wavenumber range. From Figure 3, it can be observed that there is less than 1${}^{\circ }$ variation in the output angle using a combination of prism and grating at specific angles mentioned above.

The variation in the output angle with respect to the wavenumber can be mentioned as a nonlinearity error. It is defined as a function of (Δθ/Δk). For the defined wavenumber space, using β of 25${}^{\circ }$, α of 35${}^{\circ }$ and d of 1600 lines/mm, the nonlinearity error is reduced from 147.0115 Δθ*μm in the case of grating, to 0.0149 Δθ*μm while using the combination of prism and grating. All the values are utilized for ZEMAX design.

## 3. The Optical Design of the Spectrometer Based on Transmission Grating

A new spectrometer has been designed using transmission grating in the ZEMAX platform to verify the previous section’s results. This spectrometer is designed based on a fold mirror, transmission grating, and a cylindrical focusing mirror. A super-luminescent laser diode (SLD) as the light source with a center wavelength of 830 nm and a bandwidth of 200 nm (8.607−6.756 μm${}^{-1}$ in k-space) is used for the simulation. The light first incidents onto the fold mirror and is then reflected towards the grating. A 1600 line/mm diffraction grating was used to disperse the broadband spectrum to the detector array. After grating, a cylindrical focusing mirror is needed to focus light onto the detector array. The initial values for the distance between the optical components are considered as follows: the distance between the fold mirror and the grating is 18 mm, the distance between the grating and the focusing mirror is 15 mm, and the radius of the focusing mirror is 50 mm. After that, Merit functions were used to optimize the angles and distances to minimize the aberration on the image plane [26].

**Figure 4:**
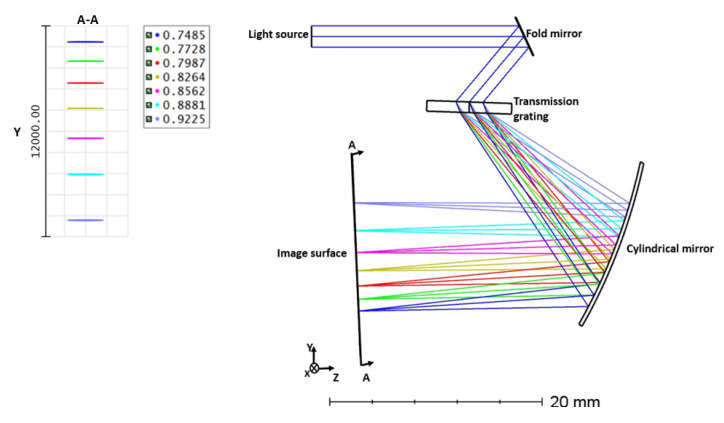
Design of the spectrometer with transmission grating in ZEMAX and dispersed spectrum on the image surface.

Each time, one of the distances and the radii are considered variable and optimized. As a result, the optimized values are 20 mm for the distance between the fold mirror and the grating, the distance between the grating and the focusing mirror is 18 mm, and the radius of the focusing mirror is 50.56 mm. The image surface used in the ZEMAX to visualize the output is similar to that of a CCD camera. In a CCD camera, the number of pixels in the line direction depends on the size of the incident beam used in the spectrometer design. In the present work, 2 mm size beams are used to evaluate the imaging performance. Figure 4 illustrates the ZEMAX design of the spectrometer and the dispersed spectrum on the image surface (CCD). For the performance analysis in ZEMAX, seven wavelengths between 730 and 930 nm are used. In Section 2, the output angle from the grating with respect to wavenumbers is sketched. Based on the simulation, it was found that the angle and wavenumbers relation is nonlinear. Similar to the results we achieved in MATLAB, the inset in Figure 4 shows the distance between each wavelength is not equal, leading to a sensitivity drop off in the depth direction.

### Spectrometer Analysis

**Figure 5:**
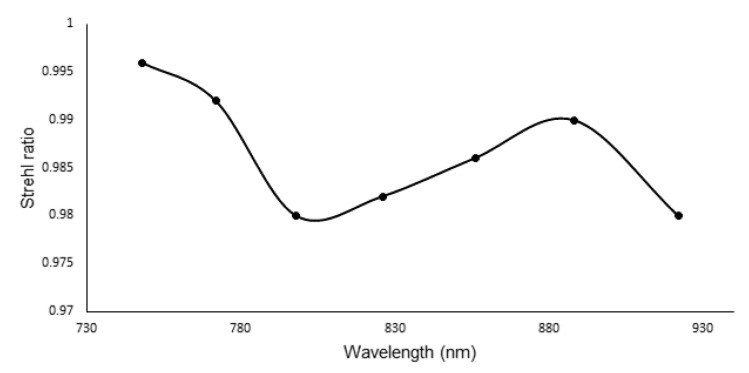
The Strehl ratio of the spectrometer with transmission grating.

The Strehl Ratio (SR) is one of the critical parameters used to evaluate the optical system’s performance. The SR is defined based on the ratio of the aberrated spread function to the aberration-free spread function. All imaging systems must meet the Marechal criterion (SR more than 0.8) to be considered an imaging system. The SR was recorded for the 2 mm beam size with 200 nm bandwidth from the ZEMAX model. The SR for the spectrometer is shown in Figure 5. Based on the SR calculations, the image resolution is high since the SR is more than 0.8 and meets the Marechal criterion. While the SR is high, the intervals of the wavelengths are nonlinear. The nonlinearity error at the output of the grating is 156.7 Δθ*μm, and this value is calculated based on the output angle of the grating in the ZEMAX simulation. The value matches closely to the nonlinearity error calculated in MATALB (147.0115 Δθ*μm). To reduce the nonlinearity error and improve transverse resolution, linearization of the wavenumbers is required.

## 4. The Optical Design of the Spectrometer Based on Transmission Grating and Prism

MATLAB modeling of the grating and prism spectrometer showed that this system would separate the wavelengths equally. The system is designed in ZEMAX to verify the linearity of the wavelengths spread on the detector, and to confirm that the focused light beam has higher SR than the Marechal criterion.

**Figure 6:**
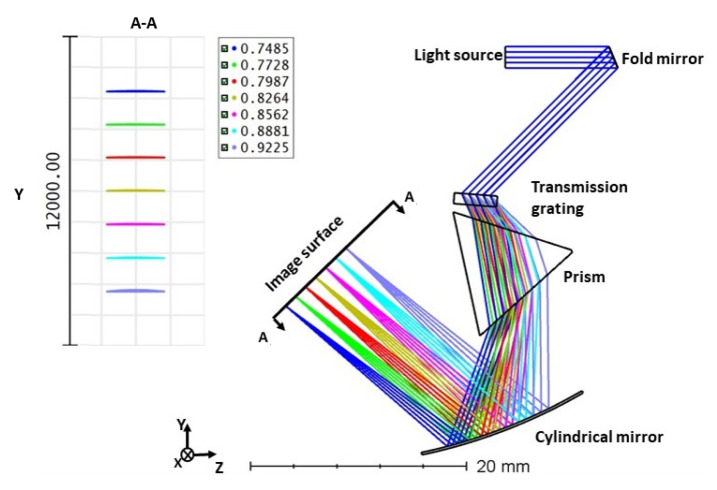
Linear k space spectrometer using grating and prism after optimization.

The same SLD from the previous design is used for this simulation as well. Seven wavenumbers (k1–k7) are chosen with an equal increment. For the design in Figure 6, we utilize the same design from Figure 4, and a prism is inserted between the grating and the cylindrical mirror. The initial values for the design are the same as the previous spectrometer. The values are optimized individually to obtain equal separation on the image surface. After optimization, the distance between the fold mirror and grating is 18 mm, the distance between the grating and the prism is 22 mm, and the radius of the cylindrical mirror is 50.56 mm. Optimization is performed to achieve equidistant dispersion of the wavelength on the image surface. The beam diffracted from the grating passes through the prism (F2 material and apex angle of 60${}^{\circ }$) and is dispersed based on the wavelength. All the wavenumbers of the dispersed light will be parallel to each other as they exit the prism. In Figure 6, it is clear that the angular variation of the wavelengths has been linearized with an error close to 0.0149 Δθ*μm.

### Spectrometer Analysis

**Figure 7:**
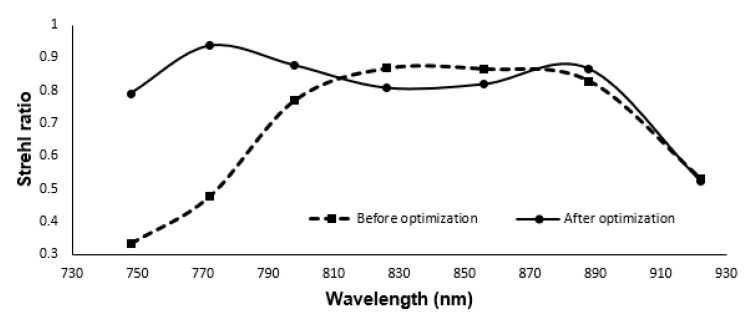
Strehl ratio of the linear spectrometer before and after optimization.

While the linearity of the wavelengths, which is the primary objective of this work, has been achieved (0.0149 Δθ*μm), the SR for four out of the seven wavelengths is significantly lower than the design without the prism. The SR for different wavelengths is shown in Figure 7 as a broken line. To improve the system’s optical quality, the distance between the cylindrical mirror and image surface, angle of the cylindrical mirror, and image surface are optimized to increase the SR. After optimization, the radius of the cylindrical mirror is 50.56 mm, and the cylindrical mirror is tilted about x, 18${}^{\circ }$. The optimized design’s Strehl ratio is shown as a solid line in Figure 7. However, the last wavelength lies below the Marechal criterion.

Though the nonlinearity error is reduced significantly, the SR ratio is still lower than the Marechal criterion for one wavelength. However, from our earlier work, we know that the all-reflective spectrometer has a higher SR ratio [23] compared to the transmission grating spectrometer. In order to improve the SR ratio while reducing the nonlinearity error, we will add a dispersive element to the all-reflective spectrometer. This design will be discussed in the next section.

## 5. The Optical Design of the Linear All-Reflective Spectrometer

**Figure 8:**
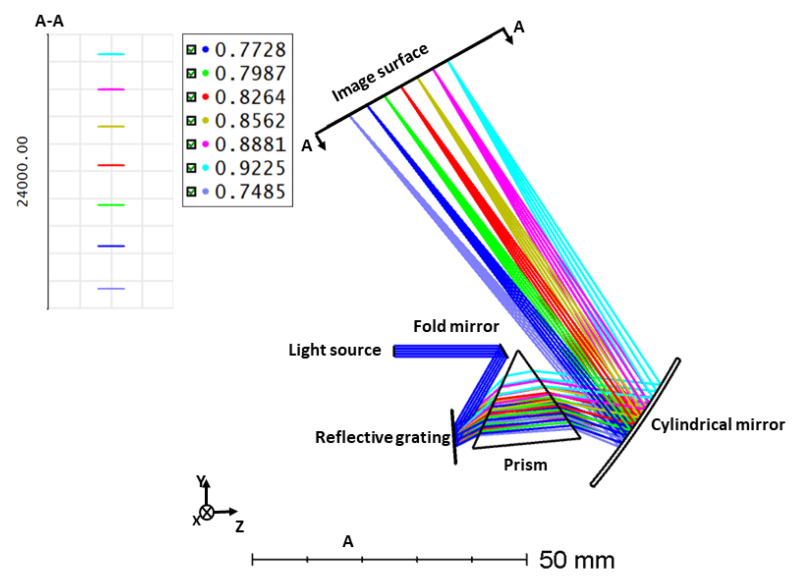
Wavenumber linear all-reflective spectrometer.

In this section, a diffraction-limited and linearized spectrometer for the center wavelength of 830 nm and spectral bandwidth of 200 nm is designed. It is composed of a fold mirror, a reflecting diffraction grating, a dispersion prism, and a concave cylindrical mirror to image the spectrum to a detector. The incoming collimated light from tissue is reflected towards the diffraction grating placed 18 mm from the fold mirror. The distance between the grating and the prism’s lower vertex is 10 mm. The beam angle to grating is $30^{\circ }$ ($\theta_{i}$). The diffracted beam is dispersed by the prism placed at an optimized angle of $32.5^{\circ }$ (β) with respect to the grating. The grating has 1600 lines/mm (d), and the prism is a commercially available $60^{\circ }$(α) wedge angle and F2 material. Merit functions are used to optimize the radius of the cylindrical mirror. A cylindrical concave mirror with a radius of 180 mm is utilized to image the dispersed beam to a CCD imaging system. Figure 8 illustrates the ZEMAX design, and the dispersion on the image surface (footprint diagram) is shown as an inset.

### 5.1. Spectrometer Analysis

**Figure 9:**
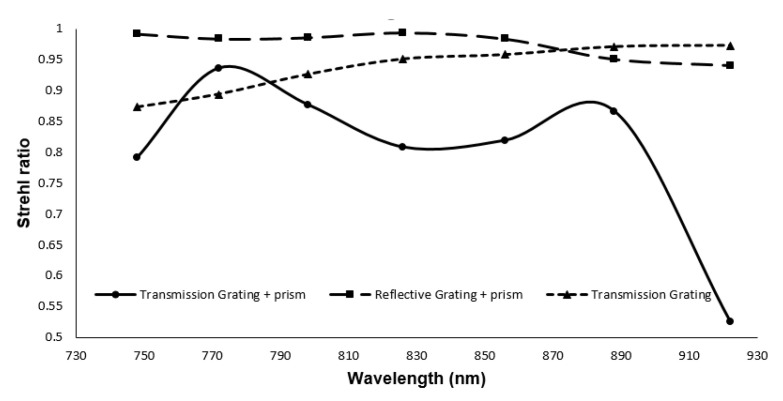
Comparison of the Strehl ratio of the linear spectrometer based on transmission grating and reflective grating.

The Strehl ratio for transmission grating and reflective grating with prism is shown in Figure 9. For comparison, it also contains the SR ratio of the transmission grating (Figure 7). From this figure, we can see that the SR ratio for the reflective grating with prism is far better than transmission grating with prism. The maximum sensitivity fall-off is −4.08 dB at 1.59 mm depth [13].

The result from the footprint diagram shows the nonlinearity error (Δθ/Δk) is 0.03517 Δθ*μm. This results in higher axial resolution and depth scan range. In our previous spectrometer design with only a grating, the nonlinearity error (Δθ/Δk) was 156.7 Δθ*μm. The Strehl ratio is above the Marechal criterion for both transmission grating and reflective grating with prism spectrometers. However, the transmission grating is not linear.

### 5.2. Comparison of the Spectrometer Design

**Figure 10:**
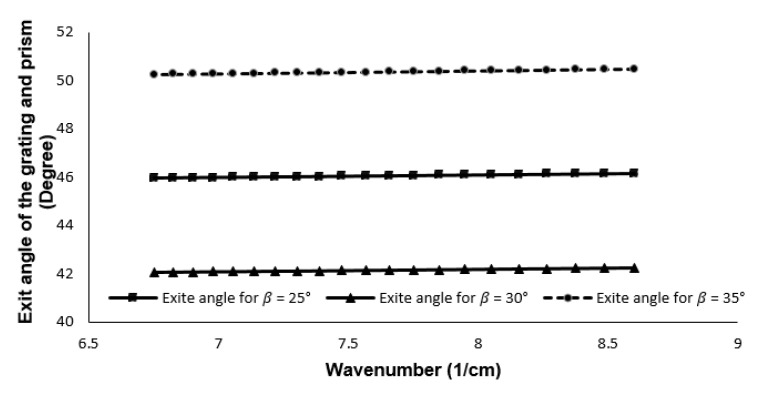
Exit angle of the grating for different β.

This work introduced different spectrometer designs for SD-OCT. For linearization, a combination of grating and prism was used. The incident angle error or a tilt between the prism and the grating may occur in this case; however, the angle between grating and prism is varied from 25${}^{\circ }$ to 35${}^{\circ }$, and the linearity is shown in Figure 10. Based on the simulation, it is obvious that the exit angle variation is less than 1${}^{\circ }$ for the ±5${}^{\circ }$ degree variation.

A summary of the designs is shown in Table 1.

## 6. Conclusions

This work introduces a linear wavenumber spectrometer with cylindrical optics for line scanning OCT. Line scanning OCT requires a spectrometer that utilizes cylindrical optics. We have demonstrated a detailed model for optimizing the dispersion components that consist of a diffraction grating and a prism. Three different spectrometers were designed. Initially, MATLAB analysis was performed to obtain the angles of the prism and grating followed by ZEMAX design. Firstly, we designed a spectrometer by transmission grating. In the second part, the transmission grating and prism are used as the dispersion group. Utilizing the dispersion group showed a considerable reduction in k-space angular nonlinearity. The nonlinearity error is reduced from 147.0115 Δθ*μm in the case of grating, to 0.0149 Δθ*μm while using the combination of prism and grating. However, the SR ratio for one wavelength was lower than the Marechal criterion. In the last part, we introduced our all-reflective spectrometer, adding a dispersive component, which shows a higher Strehl ratio compared to transmission grating (above 0.95) and maintained the low nonlinearity error (0.03517 Δθ*μm). Reducing the nonlinearity error can lead to high axial resolution and reduces the signal sensitivity fall off.

## Figures and Tables

**Table 1:** Summary of the designed spectrometers.

Spectrometer Design	Wavelength (nm)	Nonlinearity Error (Δθ/Δk)	SR Ratio
Transmission Grating	730–930	147.0115	0.98–0.996
Transmission grating and prism	730–930	0.0149	0.55–0.95
Reflective grating and prism	730–930	0.03517	0.95–0.98

## Data Availability

Not applicable.
